# The crossroads between osteosarcopenia and intrinsic capacity—a narrative review

**DOI:** 10.1093/gerona/glag102

**Published:** 2026-04-16

**Authors:** Nailton J Neto, Guy Hajj-Boutros, Wayne Lok Ok Choo, Miguel G Borda, Konstantinos Prokopidis, Hassan Rammal, Daniel Rivas, Hussein Samhat, Ivan Baltasar-Fernandez, Mahdi Imani, Cristiano dos Santos Gomes, Oscar Rosas Carrasco, Marc Sim, Julie A Pasco, Julia Chabot, Ana M Ayala-Copete, Francisco J García-García, Claire Godard-Sebillotte, Raman Agnihotram, Lana J Williams, Anna Andrianova, Robinson Ramirez-Vélez, Emma Connolly, Harmehr Sekhon, Hidenori Arai, Liang-Kung Chen, Andréa Faust, Howard Bergman, Alexandra Papaioannou, Pierrette Gaudreau, Tiago d S Alexandre, Leocadio Rodriguez-Mañas, Ricardo Oliveira Guerra, Gustavo Duque, Transforming Health and Resilience for Healthy Lives (THRIVE) Project Group

**Affiliations:** Graduate Program in Health Sciences, Federal University of Rio Grande do Norte, Natal, Brazil; Bone, Muscle & Geroscience Group, Research Institute of the McGill University Health Centre, Montreal, Quebec, Canada; RUISS McGill Simone & Edouard Schouela RUISSS McGill Centre of Excellence for Sustainable Health of Seniors (Schouela CEDurable), Montréal, Quebec, Canada; Bone, Muscle & Geroscience Group, Research Institute of the McGill University Health Centre, Montreal, Quebec, Canada; RUISS McGill Simone & Edouard Schouela RUISSS McGill Centre of Excellence for Sustainable Health of Seniors (Schouela CEDurable), Montréal, Quebec, Canada; Bone, Muscle & Geroscience Group, Research Institute of the McGill University Health Centre, Montreal, Quebec, Canada; Division of Geriatric Medicine, Department of Medicine, McGill University, Montreal, Quebec, Canada; Centre for Age-Related Medicine (SESAM), Stavanger University Hospital, Stavanger, Norway; Universidad Publica de Navarra, Navarrabiomed, Ciencias de la Salud, Pamplona, Navarra, Spain; Bone, Muscle & Geroscience Group, Research Institute of the McGill University Health Centre, Montreal, Quebec, Canada; Department of Musculoskeletal and Ageing Science, Institute of Life Course and Medical Sciences, University of Liverpool, Liverpool, United Kingdom; Bone, Muscle & Geroscience Group, Research Institute of the McGill University Health Centre, Montreal, Quebec, Canada; Bone, Muscle & Geroscience Group, Research Institute of the McGill University Health Centre, Montreal, Quebec, Canada; RUISS McGill Simone & Edouard Schouela RUISSS McGill Centre of Excellence for Sustainable Health of Seniors (Schouela CEDurable), Montréal, Quebec, Canada; GENUD Toledo Research Group, Faculty of Sport Sciences, University of Castilla-La Mancha, Toledo, Spain; Faculty of Health Sciences, University of Castilla-La Mancha, Talavera de la Reina, Spain; Grupo Mixto de Fragilidad y Envejecimiento Exitoso UCLM-SESCAM, Universidad de Castilla-La Mancha-Servicio de Salud de Castilla-La Mancha, IDISCAM, Toledo, Spain; Bone, Muscle & Geroscience Group, Research Institute of the McGill University Health Centre, Montreal, Quebec, Canada; RUISS McGill Simone & Edouard Schouela RUISSS McGill Centre of Excellence for Sustainable Health of Seniors (Schouela CEDurable), Montréal, Quebec, Canada; Department of Physiotherapy, University of Pernambuco, Petrolina, Brazil; Geriatric Assessment Center, Health Department, Iberoamerican University, Mexico City, Mexico; Nutrition & Health Innovation Research Institute, Edith Cowan University, Joondalup, Western Australia, Australia; Deakin University, School of Medicine, Deakin IMPACT-The Institute for Mental and Physical Health and Clinical Translation, Geelong, Victoria, Australia; Division of Geriatric Medicine, Department of Medicine, McGill University, Montreal, Quebec, Canada; Bone, Muscle & Geroscience Group, Research Institute of the McGill University Health Centre, Montreal, Quebec, Canada; Geriatric Medicine, Pontificia Universidad Javeriana, Bogotá, Colombia; GENUD Toledo Research Group, Faculty of Sport Sciences, University of Castilla-La Mancha, Toledo, Spain; Faculty of Health Sciences, University of Castilla-La Mancha, Talavera de la Reina, Spain; Grupo Mixto de Fragilidad y Envejecimiento Exitoso UCLM-SESCAM, Universidad de Castilla-La Mancha-Servicio de Salud de Castilla-La Mancha, IDISCAM, Toledo, Spain; Division of Geriatric Medicine, Department of Medicine, McGill University, Montreal, Quebec, Canada; Bone, Muscle & Geroscience Group, Research Institute of the McGill University Health Centre, Montreal, Quebec, Canada; Deakin University, School of Medicine, Deakin IMPACT-The Institute for Mental and Physical Health and Clinical Translation, Geelong, Victoria, Australia; RUISS McGill Simone & Edouard Schouela RUISSS McGill Centre of Excellence for Sustainable Health of Seniors (Schouela CEDurable), Montréal, Quebec, Canada; Universidad Publica de Navarra, Navarrabiomed, Ciencias de la Salud, Pamplona, Navarra, Spain; Department of Clinical Medicine, Trinity College, Dublin, Ireland; Division of Geriatric Medicine, Department of Medicine, McGill University, Montreal, Quebec, Canada; National Center for Geriatrics and Gerontology, President Office Obu, Aichi, Japan; Center for Geriatrics and Gerontology, Taipei Veterans General Hospital, Taipei City, Taiwan; Bone, Muscle & Geroscience Group, Research Institute of the McGill University Health Centre, Montreal, Quebec, Canada; RUISS McGill Simone & Edouard Schouela RUISSS McGill Centre of Excellence for Sustainable Health of Seniors (Schouela CEDurable), Montréal, Quebec, Canada; Division of Geriatric Medicine, Department of Medicine, McGill University, Montreal, Quebec, Canada; Department of Medicine, McMaster University, Hamilton, Ontario, Canada; Department of Medicine, University of Montreal, Montreal, Quebec, Canada; Research Center, Centre Hospitalier de l’Université de Montréal, Montreal, Quebec, Canada; Department of Gerontology, Federal University of Sao Carlos, Sao Carlos, Brazil; Centro de Investigación Biomédica en Red Fragilidad y Envejecimiento Saludable (CIBERFES), Instituto de Salud Carlos III, Madrid, Spain; Graduate Program in Health Sciences, Federal University of Rio Grande do Norte, Natal, Brazil; Bone, Muscle & Geroscience Group, Research Institute of the McGill University Health Centre, Montreal, Quebec, Canada; RUISS McGill Simone & Edouard Schouela RUISSS McGill Centre of Excellence for Sustainable Health of Seniors (Schouela CEDurable), Montréal, Quebec, Canada; Division of Geriatric Medicine, Department of Medicine, McGill University, Montreal, Quebec, Canada

**Keywords:** Functional ability, Musculoskeletal diseases, Aging, Sarcopenia, Osteoporosis

## Abstract

Intrinsic capacity (IC) is defined as the composite of physical and mental abilities an individual possesses, encompassing 5 domains: cognition, psychological health, sensory function, vitality, and locomotion. This construct is central to the World Health Organization’s framework for assessing functional ability in older adults. Growing evidence highlights the critical role of the musculoskeletal system in maintaining these domains, while conditions such as sarcopenia, osteoporosis, and their coexistence as osteosarcopenia (OS) are increasingly associated with IC decline. This narrative review compiles current evidence on the modulatory role of muscles and bones in IC and the impacts of sarcopenia, osteoporosis, and OS. Most findings suggest that musculoskeletal tissues influence IC not only through biomechanical functions but also as secretory organs, releasing myokines and osteokines with endocrine, paracrine, and autocrine effects. Among the most studied are brain-derived neurotrophic factor, irisin, osteocalcin, and interleukin-6. Dysregulation of these pathways, along with biomechanical dysfunction and systemic inflammation, links sarcopenia, osteoporosis, and OS to IC impairment. Further research is needed to clarify the specific mechanisms involved, particularly in the sensory and vitality domains, to inform targeted interventions that promote healthy aging.

## Introduction

### Sarcopenia, osteoporosis, and osteosarcopenia

Sarcopenia and osteoporosis are two common musculoskeletal conditions in older adults, defined as the progressive loss of muscle mass and strength,^1^ and by reduced bone mass and microarchitectural deterioration,^2^ respectively. According to different diagnostic criteria, sarcopenia affects 10%-27% of this population globally,^3^ while pooled estimates suggest a global prevalence of osteoporosis ranging from 21% to 35% among older adults.^4^ The substantial variability in the reported prevalence of sarcopenia and osteoporosis is largely attributable to heterogeneity in diagnostic definitions, assessment methods, and population characteristics, especially in sarcopenia.^5^ Both conditions are linked to higher rates of hospitalization, disability, fractures, morbidity, and mortality.^6^^,^^7^

With aging, bone and muscle undergo common changes at both macroscopic and molecular levels, including altered biomechanical interactions and secretion of osteokines and myokines.^8^ Muscle-bone crosstalk deteriorates, in part, when muscle weakness reduces osteogenic mechanical stimuli, promoting loss of bone mass and quality. In addition, reduced bone mineral density (BMD) compromises the structural integrity of the skeleton, increasing fragility and functional limitation, which can exacerbate physical inactivity and accelerate muscle loss.^9^ On the one hand, sarcopenia increases the risk of osteoporosis by approximately 3 times, while osteoporosis has been associated with a 2-fold increase in the risk of sarcopenia.^10^ Thus, the coexistence of these conditions has become the focus of research in geriatrics and gerontology, recognized as a new geriatric syndrome called osteosarcopenia (OS).^11^

OS arises from disrupted physiological interactions between bones and muscles and is estimated to affect 18%-21% of older adults,^12^ with higher rates observed in women and individuals aged ≥80 years. The main risk factors include female gender, advanced age, and previous fractures.^13^ Its pathophysiology involves genetic predisposition, reduced mechanical loading, and endocrine dysfunction, particularly low levels of growth hormone (GH), insulin-like growth factor-1 (IGF-1), and sex hormones.^14^ GH and IGF-1 support bone formation by stimulating osteoblasts while also promoting muscle anabolism.^15^ Similarly, estrogen receptors, androgen metabolites, and testosterone play key roles in preserving musculoskeletal integrity.^16^^,^^17^

### Intrinsic capacity and OS

Intrinsic capacity (IC) describes the composite of an individual’s physical and mental capacities and is central to the World Health Organization’s (WHO) framework for healthy aging.^18^^,^^19^ Introduced in the *2015 World Report on Ageing and Health*, IC emphasizes functional ability over disease-centered models of care.^20^ Cesari et al.^21^ identified 5 domains of IC: *cognition* (memory, learning, decision-making), *psychological health* (mood, depressive symptoms, emotional vitality), *sensory function* (vision and hearing), *vitality* (energy metabolism, neuromuscular and immune functions, homeostasis), and *locomotion* (gait, balance, strength, mobility).^21–23^ These domains were derived from risk factors consistently associated with functional decline in older adults.^24^^,^^25^ An important methodological consideration is the potential overlap between IC and the operational definitions of sarcopenia and frailty. Sarcopenia definitions often include grip strength and gait speed, which align closely with the locomotion domain of IC, and frailty may share similar components depending on the model used. This shared measurement should be kept in mind when interpreting associations between OS and IC, as it may contribute to stronger observed relationships in physical domains. IC predicts changes in function, frailty, and falls,^26^ outcomes closely tied to musculoskeletal health. Declines in muscle strength and quality reduce mobility, gait speed, and balance,^27^ while osteoporotic vertebral fractures exacerbate limitations through pain and postural deformities, encouraging sedentary behavior and accelerating decline.^28^ Thus, locomotion is the domain most directly affected, though other domains are also influenced. Emerging studies link sarcopenia and osteoporosis to IC decline. Zhu et al.^29^ reported that both conditions independently impair IC in hospitalized older adults. Ling et al. showed that probable sarcopenia is strongly associated with increased dementia risk,^30^ while osteoporosis has also been linked to cognitive impairment.^31^ Importantly, the coexistence of both conditions amplifies risk, as Inoue et al. demonstrated: OS was associated with higher rates of both functional and cognitive impairment than sarcopenia or osteoporosis alone.^32^ Muscles and bones act as endocrine organs, releasing myokines and osteokines that regulate physiological processes beyond their tissues of origin.^33^^,^^34^ Effective secretion of these factors is essential to maintain IC, while conditions such as OS disrupt these signals, driving systemic inflammation and dysregulation.^8^ This contributes to reduced physiological reserve, greater vulnerability, and loss of autonomy.

To date, no review study has specifically examined the impact of OS on individual domains of IC. However, a recent systematic review by Long et al.^35^ assessed the association between OS and frailty in older adults. Although this review was not specifically designed to examine IC, the multidimensional nature of frailty suggests a conceptual and clinical overlap with IC domains. In particular, components of frailty syndrome, such as reduced physical performance, decreased energy and endurance, cognitive impairment, and psychosocial vulnerability, may plausibly correspond to declines in the locomotion, vitality, cognition, and psychological domains of IC. Therefore, findings from frailty-based frameworks may provide indirect evidence supporting the relevance of OS to IC trajectories in older people. Thus, this narrative review aims to discuss how musculoskeletal changes influence IC, focusing on factors derived from muscles and bones, and to highlight the combined effects of sarcopenia, osteoporosis, and OS in the domains of IC.

## Methods

### Eligibility criteria

This narrative review included studies involving adults (men and women) aged ≥65 years that examined OS, osteoporosis, or sarcopenia and assessed at least 1 of the 5 IC domains. Eligible designs were observational, experimental, preclinical, or review articles, provided they reported outcomes or mechanisms relevant to IC. Articles identified through reference lists were also considered. Studies were excluded if they did not evaluate IC or its domains.

### Searches

Literature searches were performed in PubMed, Embase, Web of Science, Scopus, and Google Scholar between February and May 2025. Search terms combined descriptors related to aging, sarcopenia, osteoporosis, osteosarcopenia, IC, and its domains, using both MeSH and free-text terms. Although the primary search covered 2015-2025, earlier relevant articles were also considered.

### Data extraction

Titles, abstracts, and full texts were reviewed to identify relevant studies. Extracted data included sample characteristics, risk estimates, and biological mechanisms linking muscle, bone, sarcopenia, osteoporosis, and OS to IC. This approach allowed integration of quantitative findings and mechanistic insights to better understand how muscle and bone health influence IC.

## Results and discussion

The following subsections examine each domain of IC (cognition, psychological, sensory, vitality, and locomotion), emphasizing how sarcopenia, osteoporosis, and OS contribute to their decline and highlight critical gaps for intervention.

### Cognition domain

Muscle and bone play a central role in cognitive health through both biomechanical and endocrine mechanisms. Myokines released during muscle contraction, such as brain-derived neurotrophic factor (BDNF), cathepsin B, insulin-like growth factor-1 (IGF-1), lactate, irisin, and fibroblast growth factor 21 (FGF-21), support neurogenesis, synaptic plasticity, angiogenesis, and energy homeostasis.^36–50^ BDNF, although primarily synthesized in the brain, is also produced by skeletal muscle and regulates adult neurogenesis and synaptic plasticity.^40^^,^^42^^,^^44^ Irisin crosses the blood-brain barrier, binds to the αVβ5 integrin receptor, and upregulates hippocampal BDNF, enhancing synaptic plasticity and memory.^36^^,^^38^^,^^39^^,^^47^^,^^48^ Lower circulating irisin levels are consistently reported in individuals with sarcopenia.^49^^,^^50^ Similarly, FGF-21, secreted in response to exercise and metabolic stress, activates PI3K-Akt and MAPK/ERK pathways and stimulates PGC-1α-mediated mitochondrial biogenesis, thereby reducing oxidative stress and preserving neuronal energy homeostasis.^37^

Motor and cognitive functions also share overlapping brain regions, including the cerebellum, basal ganglia, hippocampus, and frontal and parietal cortices.^51–53^ The hippocampus is particularly important, as its grid, place, and speed cells integrate spatial orientation and locomotor control, reinforcing the motor-cognition link.^54^^,^^55^

Sarcopenia significantly increases the risk of cognitive decline. Ceccarelli et al.^56^ showed that sarcopenia impairs cognition by disrupting myokine secretion, inflammation, and reducing glucose storage. Pro-inflammatory cytokines crossing the blood-brain barrier are believed to affect memory.^57^ Mendelian randomization (MR) suggests a bidirectional causality: low appendicular muscle mass (*β* = .07; 95% CI 0.04-0.11) and slow gait speed (*β* = .57; 95% CI 0.38-0.77) are linked to cognitive decline,^58^ with further evidence that reductions in muscle strength and physical function mediate this relationship.^59^ Another MR study showed that a one standard deviation decrease in handgrip strength, appendicular lean mass, whole-body lean mass, and gait speed increases Alzheimer’s disease risk, with risk ratios ranging from 1.10 (95% CI 1.05-1.15) to 1.28 (95% CI 1.19-1.38).^60^ Meta-analyses confirm the strength of this association. The prevalence of mild dementia among individuals with sarcopenia is estimated at 20.5%.^61^ The odds of cognitive impairment are nearly threefold higher in sarcopenic adults (unadjusted OR 2.93, 95% CI 2.30-3.73; adjusted OR 2.25, 95% CI 1.21-4.17).^62^ Functional indicators of sarcopenia, slower gait speed, and weaker grip strength are also independently associated with cognitive decline.^63–65^ Mechanistically, reductions in BDNF, IGF-1, IL-6, and irisin likely contribute to neurodegeneration,^46^ while vascular dysfunction, including carotid atherosclerosis and impaired endothelial function, further links sarcopenia to memory-related deficits.^57–59^ Shared factors such as oxidative stress, insulin resistance, and lower sex steroid levels also underlie the parallel decline of muscle and cognition.^51^^,^^66^^,^^67^

Bone-derived hormones also influence cognition. Undercarboxylated osteocalcin (uOCN) crosses the blood-brain barrier, prevents hippocampal apoptosis, and regulates neurotransmitter synthesis.^68–70^ Low serum uOCN is associated with structural brain changes and poorer cognitive performance.^71–73^ Other osteokines, such as periostin and sclerostin, influence cortical plasticity and neurogenesis via Wingless-related integration site protein (Wnt)/β-catenin signaling.^74–77^ Clinically, osteoporosis nearly doubles the risk of cognitive impairment (meta-analysis OR 2.01, 95% CI 1.63-2.48).^31^ Mechanisms include hypothalamic-pituitary-adrenal (HPA) axis dysregulation—higher cortisol levels in osteoporotic women correlate with worse declarative memory^78^—vitamin D deficiency,^79^ and cerebrovascular disease.^80^ Elevated Dickkopf-related protein 1 (DKK1), a Wnt antagonist, is linked to both osteoporosis and synaptic dysfunction.^81^^,^^82^

Low BMD is another marker of cognitive risk. A large cohort of 8618 women confirmed that low BMD increased the risk of all-cause dementia (HR 1.58, 95% CI 1.20-2.08) and Alzheimer’s disease (HR 1.61, 95% CI 1.11-2.36).^83^^,^^84^ Reduced femoral neck BMD increases the risk of Alzheimer’s disease (HR 2.19, 95% CI 1.67-2.88),^84^ and BMD predicts learning ability in middle-aged adults.^85^ Conversely, higher BMD is protective: dementia risk decreases with greater BMD at the lumbar spine (HR 0.85, 95% CI 0.76-0.95), trochanter (HR 0.78, 95% CI 0.68-0.90), and total femur (HR 0.82, 95% CI 0.72-0.93).^86^

The combined effects of sarcopenia and osteoporosis appear synergistic. Inoue et al.^32^ studied 432 frailty-clinic patients and found that 10.2% had OS and 20.8% cognitive frailty, a condition of decreased cognitive reserve, considered to coexist with mild cognitive impairment (MCI) and physical frailty.^87^ OS was strongly associated with cognitive frailty (OR 8.24, 95% CI 3.31-20.48), compared to sarcopenia (OR 4.89, 95% CI 2.03-11.77) or osteoporosis alone (OR 2.16, 95% CI 1.03-4.54). OS was also linked to impairments in visuospatial/executive function and orientation (OR 3.27, 95% CI 1.16-9.24).

**Figure 1:**
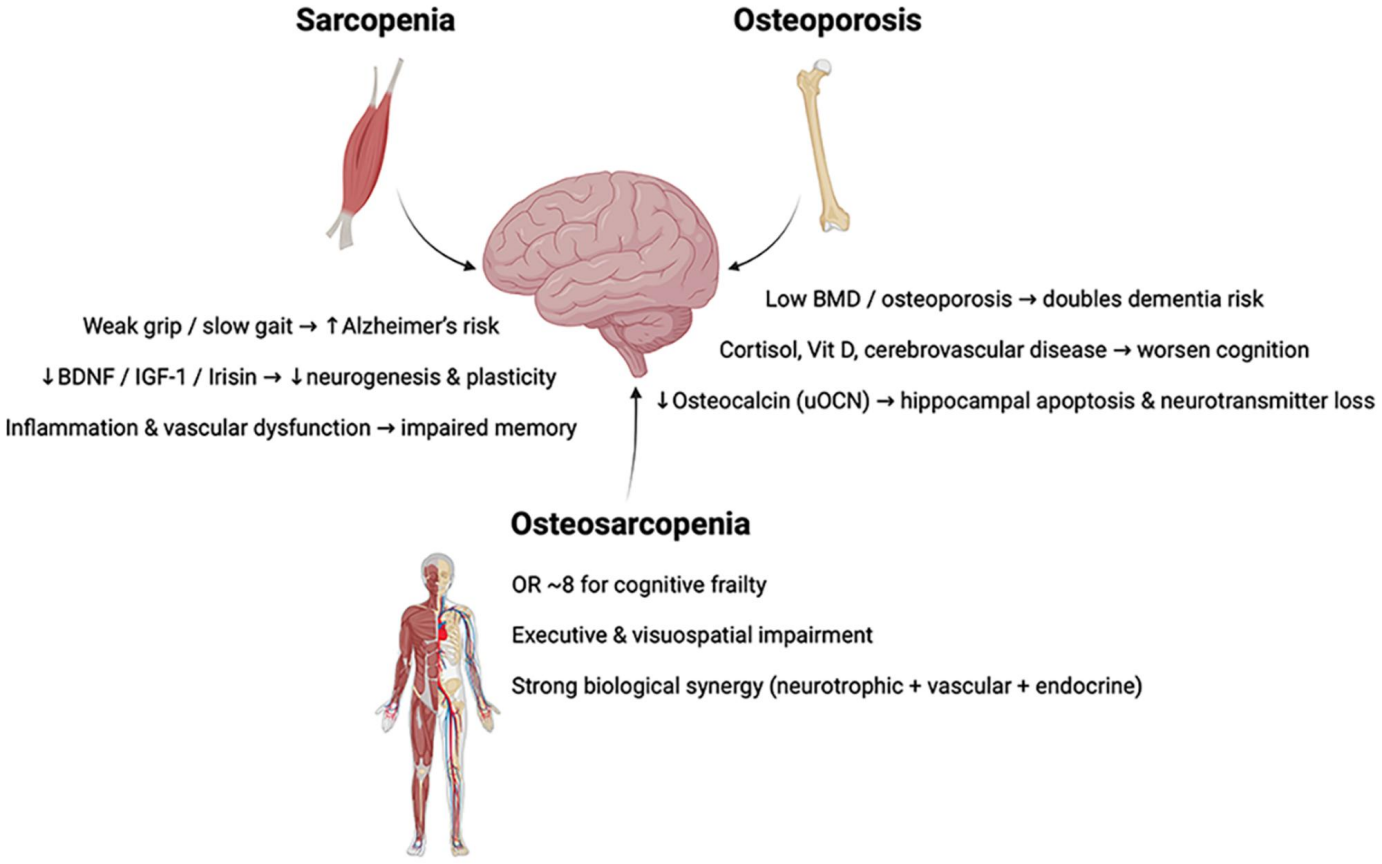
Declines in cognition domain due to sarcopenia, osteoporosis, and osteosarcopenia. BDNF: brain-derived neurotrophic factor; IGF-1: insulin-like growth factor 1; BMD: bone mineral density; vit D: vitamin D; OR: odds ratio.

In summary, muscle- and bone-derived endocrine factors such as BDNF, irisin, FGF-21, and osteocalcin strongly influence cognitive resilience. Sarcopenia and osteoporosis each impair cognition through inflammation, vascular dysfunction, hormonal dysregulation, and reduced neurotrophic signaling (Figure 1). Their coexistence as OS magnifies these risks, with effect sizes substantially greater than either condition alone.

### Psychological domain

Psychological health in aging is closely influenced by musculoskeletal status through endocrine, inflammatory, and psychosocial mechanisms. Muscle-derived BDNF promotes hippocampal neurogenesis and stress regulation, and its secretion is modulated by exercise and nutritional status.^88–90^ Downregulation of the PGC-1α-FNDC5-BDNF pathway in skeletal muscle has been shown in animal models to increase vulnerability to stress and depressive behaviors.^91^ Other myokines also contribute: acutely released IL-6 exerts anti-inflammatory and neuroprotective effects,^92–94^ while chronic elevations of IL-1, TNF-α, CRP, and IL-6 are strongly linked to depression.^95^ Irisin, which influences mood and resilience, is often reduced in sarcopenic individuals.^93^

Higher muscle strength and function have been consistently associated with a lower risk of depression. In a large cohort study, Gu et al.^96^ found that those with the highest handgrip strength had a 56% lower risk of major depressive disorder (HR = 0.44; 95% CI 0.39-0.50) compared with those with the lowest. This protective effect remained after adjusting for age, sex, BMI, physical activity, and genetic risk, indicating that muscle strength can buffer against genetic predisposition. MR studies reinforce these findings: higher hand grip strength (HGS) (right: OR = 0.88, 95% CI 0.79-0.99; left: OR = 0.81, 95% CI 0.73-0.91) and faster gait speed (OR = 0.67, 95% CI 0.51-0.90) are causally associated with lower depression risk.^97^ Clinically, sarcopenia correlates strongly with depressive symptoms (*β* = .663),^98^ while slower performance on the Five Times Sit-to-Stand test predicts depression longitudinally (HR = 1.32, 95% CI 1.08-1.62). Conversely, higher muscle strength is protective (HR = 0.46, 95% CI 0.30-0.69).^99^ Mechanistically, sarcopenia may lower BDNF and irisin availability, impair mobility, reduce independence, and increase social isolation, all recognized drivers of mood disorders.^99^

Bone-derived hormones also regulate mood. uOCN reduces anxiety-like behaviors in mice by binding to GPR158 in the hippocampus and prefrontal cortex,^69^^,^^100^ while deficiencies are linked to anxiety and depressive phenotypes. Vitamin D, acting through adrenal cortex receptors, protects against dopamine and serotonin depletion and regulates the hypothalamic-pituitary-adrenal axis.^101^^,^^102^ In older adults, osteoporosis contributes to mood disorders via psychosocial and physical pathways: fear of falling, chronic pain (especially from vertebral fractures), hyperkyphosis, and postural deformities lower self-esteem, impair body image, and foster anxiety and sadness.^103–107^ These limitations restrict participation in enjoyable activities, increase dependency on caregivers, and add financial stress, further elevating risk for depression.^108^

OS appears to amplify these risks. Blomqvist et al.^109^ reported that OS was associated with significantly greater depressive symptoms than sarcopenia alone (OR = 2.75, 95% CI 1.63-4.64) in a cohort of 2142 adults ≥55 years. Park et al.^110^ found that 19.2% of 885 individuals with OS scored higher on the GDS-SF-K (mean = 4.6, 95% CI 3.9-5.4) compared with those with sarcopenia or osteopenia alone. In the SHARE study (n = 16 452), Veronese et al.^111^ confirmed that OS was associated with a higher risk of depression over 12 years (HR = 1.27, 95% CI 1.12-1.58). These associations likely reflect the combined impact of muscle weakness and bone fragility on mobility, autonomy, and psychosocial well-being, alongside chronic inflammation and hormonal imbalances.

**Figure 2:**
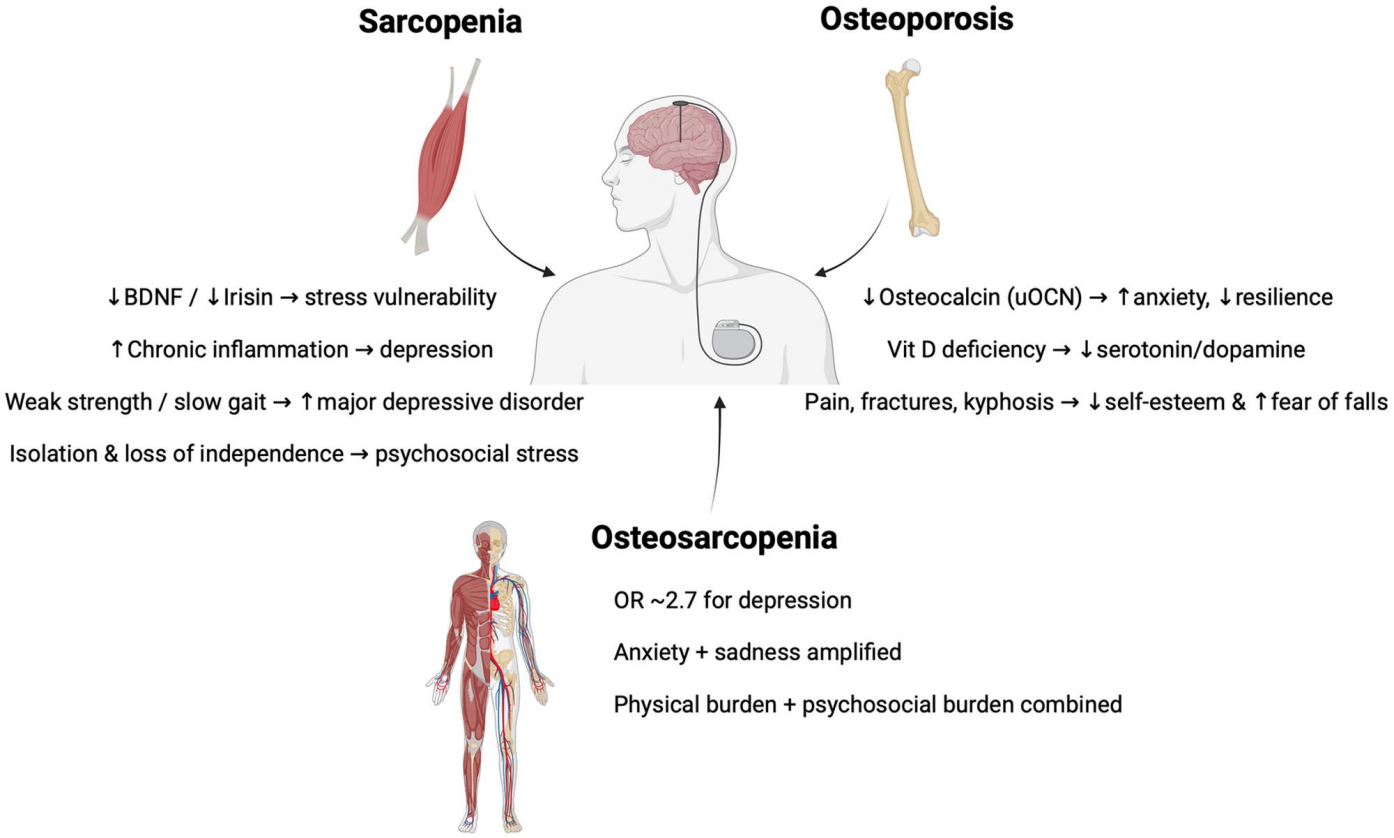
Declines in the psychological domain due to sarcopenia, osteoporosis, and osteosarcopenia. BDNF: brain-derived neurotrophic factor; vit D: vitamin D; OR: odds ratio.

In summary, sarcopenia promotes depression via loss of strength, reduced BDNF and irisin, and increased isolation, while osteoporosis contributes through chronic pain, postural deformities, fear of falls, and reduced independence. When both conditions coexist as OS, the risk of depression and emotional decline is substantially higher than either condition alone (Figure 2). Addressing OS therefore, requires not only physical rehabilitation but also psychological support to maintain emotional vitality in older adults.

### Sensory domain

Evidence linking musculoskeletal health to sensory function, particularly hearing and vision, is emerging and increasingly suggestive of meaningful associations.

#### Hearing

In a prospective cohort of 21 907 adults followed for 6 years, higher muscular fitness was associated with a 21% lower risk of hearing loss (HR = 0.79; 95% CI 0.71-0.88), with protective effects seen across handgrip strength, vertical jump, balance, flexibility, and reaction time.^112^ Mechanistically, muscle contraction contributes to auditory protection via the acoustic reflex, where the tensor tympani and stapedial muscles reduce sound transmission in response to loud stimuli.^113–115^ More broadly, muscle mass influences cochlear function by regulating cardiovascular demand and cochlear perfusion, as the stria vascularis is highly vascularized and sensitive to changes in blood flow.^116–122^

Sarcopenia appears to increase hearing loss risk through vascular and metabolic pathways. MR analysis^123^ demonstrated that slower walking pace increased risk of sensorineural hearing loss (OR = 0.53; 95% CI 0.30-0.92), weaker HGS was associated with conductive hearing loss (OR = 0.41; 95% CI 0.21-0.80), and lower appendicular lean mass increased mixed hearing loss risk in women (OR = 0.84; 95% CI 0.73-0.98). Sarcopenia promotes arteriole stiffening,^124^^,^^125^ oxidative stress,^126^ and metabolic disorders such as diabetes and hyperlipidemia, which damage the stria vascularis and impair ion transport, ultimately leading to sensorineural hearing loss.^127–130^

Osteoporosis also impacts auditory function. The ossicles (malleus, incus, stapes) require proper mineralization for sound transmission.^131–134^ Osteocyte mechanotransduction is disrupted in osteoporosis, resulting in reduced mineralization and compromised conduction.^135–137^ A meta-analytic study found that low BMD increased hearing loss risk (OR = 1.20; 95% CI 1.01-1.42).^138^ Mechanistic explanations include demineralization of the petrous temporal bone, weakening of the cochlear capsule, and degeneration of stereocilia.^139–142^ Additional processes involve hyalinization of cochlear ligaments^143^ and disrupted ion metabolism due to altered bone turnover.^138^ In mice, OPG deficiency causes both sensorineural and conductive hearing loss through cochlear nerve degeneration, ERK pathway activation, and apoptosis of spiral ganglion cells.^144^^,^^145^

#### Vision

Evidence is less robust but suggests a potential role of musculoskeletal health in ocular function. The ciliary muscle enables accommodation by adjusting lens curvature; however, its mobility decreases with aging due to structural stiffening of ocular tissues.^146–148^ Sarcopenia may contribute indirectly: visual impairment has been associated with lower muscle mass and slower gait speed,^149^ and ciliary dysfunction may accelerate presbyopia.^147^^,^^150^ Experimental studies also suggest that muscle-derived DEL-1 may protect retinal pigment epithelial cells against stress-induced apoptosis, potentially reducing the risk of age-related macular degeneration (AMD).^151^ Bone metabolism may also contribute to vision. Abnormal calcium and phosphate release due to low BMD could promote ectopic calcification. Hydroxyapatite (HAP) spheres, found in Bruch’s membrane of aging eyes, nucleate drusen formation and block metabolic exchange between the retina and choroidal capillaries.^152–154^ This process, implicated in AMD progression, may be exacerbated by osteoporosis-related mineral imbalances.^155^^,^^156^

Although direct evidence is scarce, OS may amplify these sensory deficits. For hearing, sarcopenia impairs cochlear perfusion and promotes metabolic dysfunction, while osteoporosis weakens the cochlear capsule and alters ion balance. Together, these mechanisms increase the risk of hearing loss. For vision, sarcopenia may accelerate presbyopia through muscle dysfunction, while osteoporosis may potentiate AMD via increased mineral deposition and HAP-driven drusen formation. Thus, OS likely heightens vulnerability to both auditory and visual decline in older adults.

**Figure 3:**
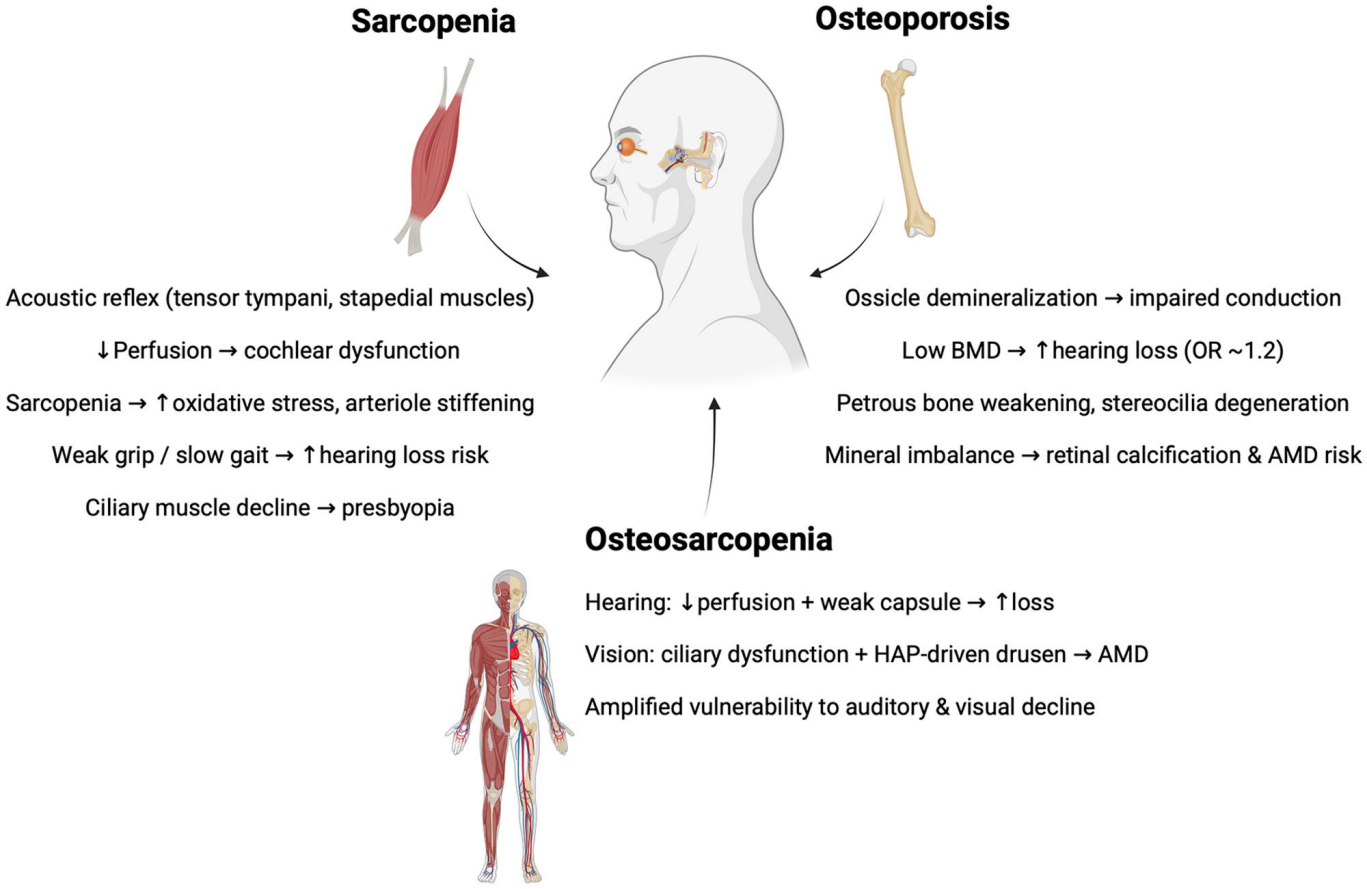
Declines in the sensory domain due to sarcopenia, osteoporosis, and osteosarcopenia. BMD: bone mineral density; HAP: hydroxyapatite; AMD: age-related macular degeneration; OR: odds ratio.

Growing evidence supports a link between OS and hearing impairment in older adults, indicating that sarcopenia and osteoporosis may both contribute to hearing decline through different but related mechanisms, as discussed earlier. Sarcopenia has been linked to several types of hearing loss, as shown in previous studies. Other mechanisms might include hyalinization of the cochlear ligaments and disrupted ion metabolism caused by imbalances in bone remodeling. Overall, these findings suggest that sarcopenia and osteoporosis, through vascular, metabolic, and structural pathways, can both contribute to hearing loss in older adults. In summary, muscle and bone integrity are increasingly recognized as modulators of sensory health (Figure 3). When combined, OS plausibly amplifies these risks, highlighting sensory decline as an emerging but understudied domain of musculoskeletal aging.

### Vitality domain

Skeletal muscle is central to the vitality domain of IC through its endocrine, metabolic, and cardiovascular functions. Myokines such as β-aminoisobutyric acid (BAIBA), myostatin, myonectin, irisin, IL-6, IL-15, BDNF, and FGF-21 regulate glucose metabolism, fatty acid oxidation, insulin sensitivity, thermogenesis, and cardiovascular health.^48^^,^^157–160^ Muscle also supports vitality via the muscle pump, enhancing circulation, oxygen delivery, and gas exchange during activity.^116^^,^^161^ Greater muscle mass correlates with higher VO_2_ max, increased endurance, and improved oxygen extraction, whereas aging-related declines in cardiac output, muscle perfusion, mitochondrial density, and oxidative enzyme activity impair oxidative metabolism.^119^^,^^162^^,^^163^

Sarcopenia compromises these functions. In a cohort of 102 older men, the sarcopenic group (14.7% of the sample) had significantly lower peak VO_2_ and peak O_2_ pulse compared to their non-sarcopenic peers.^162^ Cardiac sarcopenia, characterized by reduced cardiomyocytes and increased fibrosis, impairs ventricular function,^160^ while respiratory muscle loss reduces inspiratory/expiratory strength, as well as lung volumes such as FVC and FEV_1_.^164^^,^^165^ MR confirms causal links: lower skeletal muscle mass (SMM) increased risk of CAD, stroke, and MI (ORs 0.81-0.93), while stronger handgrip protected against CAD and MI.^166^

Handgrip strength is widely validated as an indicator of vitality, predicting functional reserve, recovery capacity, and adverse outcomes.^167^^,^^168^ Sarcopenia-related declines in HGS reflect chronic inflammation (IL-6, TNF-α),^169^ protein degradation via the ubiquitin-proteasome system,^170^^,^^171^ oxidative stress,^172^ and hormonal deficits.^173^ Neuromuscular junction degeneration, cortical hypoexcitability, fiber-type shifts (loss of type II fibers), and HGS asymmetry (>10% between hands) further weaken performance.^174–177^ Nutrition also links sarcopenia to vitality: meta-analytic evidence indicates that sarcopenic adults have significantly lower intakes of protein, vitamins A, B12, C, D, selenium, and essential minerals.^178^ Sarcopenic dysphagia, due to weakening of swallowing muscles, worsens nutrient intake and perpetuates muscle loss.^179–181^ Together, these mechanisms create a negative feedback loop of poor nutrition, muscle decline, and reduced vitality.

Bone also contributes via osteokines. uOCN improves glucose tolerance, β-cell function, and insulin sensitivity, while higher circulating levels correlate with lower adiposity and BMI.^182^^,^^183^ Lipocalin-2 (LCN2), secreted by osteoblasts, enhances glucose tolerance and energy expenditure, crosses the blood-brain barrier to act on hypothalamic MC4R pathways, and suppresses appetite.^184–187^ Clinically, LCN2 has been linked to insulin sensitivity and β-cell function, paralleling uOCN.^188^ These data support bone as an active regulator of metabolism and energy balance.

Although the available evidence on the associations between osteoporosis and vitality, and its subdomains, is limited, previous analyses indicate that osteoporosis further impairs vitality through its association with frailty. An MR study found that osteoporosis increased frailty risk (OR = 2.81, 95% CI 1.69-4.68), mediated by hormonal decline and chronic inflammation.^189^ Low BMD is linked to respiratory sarcopenia: in 530 older adults, those in the lowest BMD tertile had over fourfold higher odds of respiratory sarcopenia (OR = 4.52, 95% CI 1.71-13.1).^164^ In the UK Biobank (>300 000 participants), men with osteoporosis had an increased risk of respiratory diseases (HR = 1.26), particularly COPD (HR = 1.82).^190^ Mechanistically, reduced uOCN impairs mitochondrial efficiency and aerobic capacity,^68^ while osteoporosis-associated vascular calcification compromises perfusion.^191^^,^^192^ Together, low BMD, lean mass, and vascular dysfunction reduce pulmonary ventilation and cardiorespiratory reserve, diminishing vitality.^193^

Although direct evidence is limited, OS likely exacerbates vitality decline by combining the metabolic and cardiorespiratory effects of sarcopenia with the frailty and inflammatory burden of osteoporosis. Shared mechanisms include chronic inflammation, hormonal dysregulation, vascular impairment, and nutritional deficits, which reinforce one another in a vicious cycle of declining functional capacity.^32^^,^^194^^,^^195^ Incorporating both HGS and BMD into geriatric assessments may therefore improve evaluation of vitality risk in older adults.

**Figure 4:**
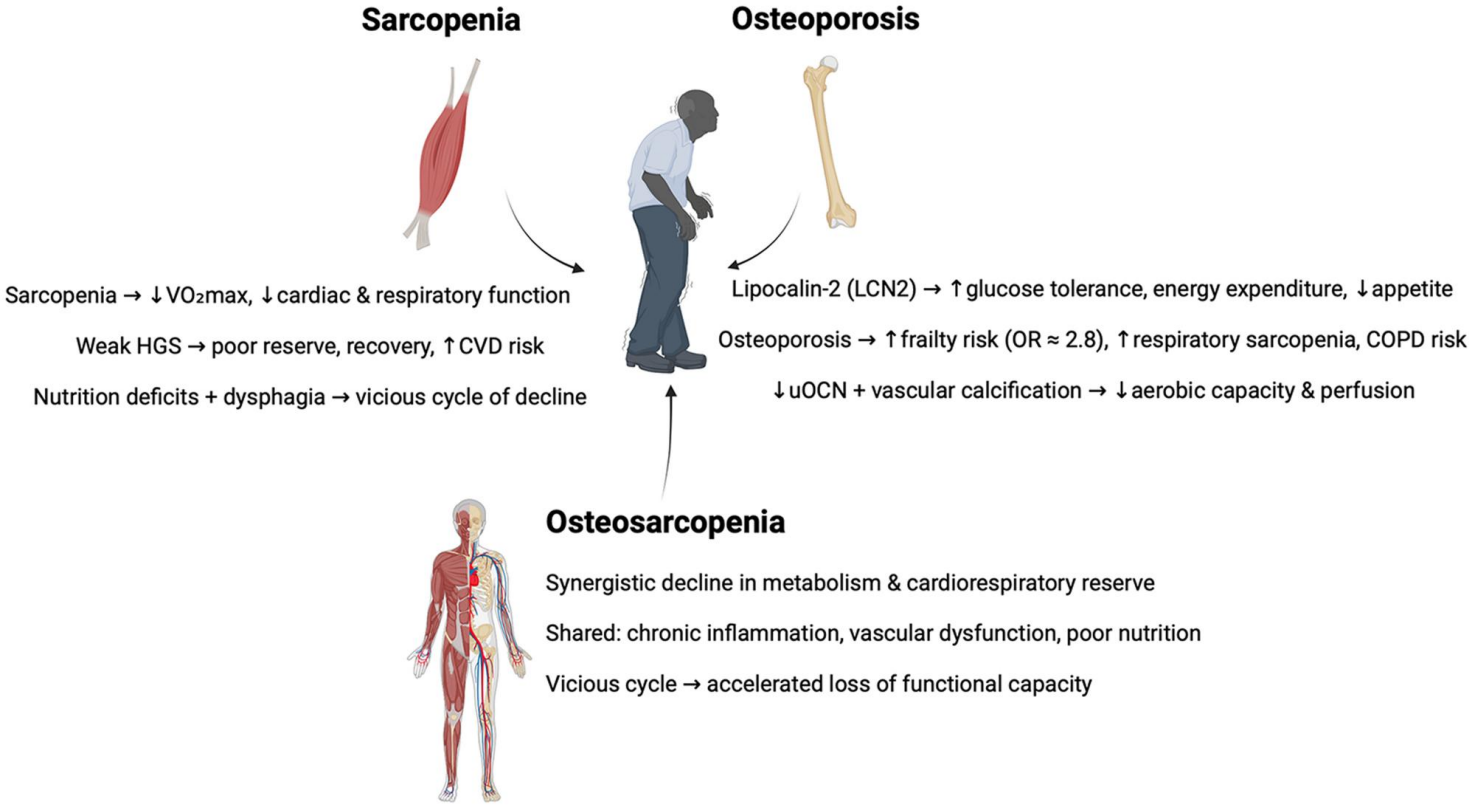
Declines in vitality domain due to sarcopenia, osteoporosis, and osteosarcopenia. uOCN: undercarboxylated osteocalcin.

In summary, skeletal muscle preserves vitality via myokine-driven regulation of energy and cardiovascular function, while sarcopenia compromises this through metabolic dysfunction, reduced strength, and poor nutrition. Bone contributes through osteokines like uOCN and LCN2, whereas osteoporosis impairs cardiorespiratory reserve and increases frailty risk. Their coexistence as OS likely accelerates these declines (Figure 4), underscoring the importance of integrated strategies to preserve vitality in aging.

### Locomotion domain

Locomotion depends on coordinated muscle actions, intact neuromuscular control, and skeletal support. In stance and gait, plantar flexors (gastrocnemius, soleus) stabilize the ankle, quadriceps/hamstrings/gluteals bear load and enable postural adjustments, while trunk and head-neck extensors provide proximal stability.^196–203^ Force generation reflects force-length/velocity properties and muscle architecture (pennation, fascicle length), with graded motor unit recruitment according to Henneman’s size principle.^204–207^ Aging disrupts these mechanisms: neuromuscular activation declines, architecture remodels, and postural/gait control deteriorate, reducing strength and increasing instability.^184^^,^^208^ Contraction-induced myokines help preserve muscle quality and, by extension, balance and mobility. IL-6 supports myogenesis acutely, but chronic elevation promotes catabolism; irisin supports hypertrophy and modulates IL-6; Secreted Protein Acidic and Rich in Cysteine (SPARC) and BDNF aid regeneration; IGF-1 activates anabolic pathways and satellite cells; IL-15 supports hypertrophy and mitochondrial function; and myonectin enhances protein synthesis.^33^^,^^42^^,^^43^^,^^209–212^ Muscle extracellular vesicles deliver IGF-1 and bFGF that facilitate repair.^213^^,^^214^ Collectively, these signals sustain the strength-mass relationship essential to static balance and functional mobility.^215^

Sarcopenia impairs gait and balance through muscular, cardiovascular, and neural pathways.^216^^,^^217^ Gait markers, shorter step/stride length, emerge early^218^^,^^219^: in 68 community-dwelling older adults (24 sarcopenic, 44 non-sarcopenic), step and stride length were significantly reduced in sarcopenia.^220^ Over 9 years in 600 older adults, transitions to slower walking speed were positively associated with IGF-1 (men), and gait speed positively correlated with LDH, creatinine, IGF-1, and appendicular muscle mass.^221^ Biomarkers also map to performance: lower TNF-α aligned with faster walking speed (0.82 vs 0.64 m/s) and lower SARC-F (1.73 vs 3.26); lower myostatin and P3NP related to faster speed (*β* = −.309) and better SPPB (*β* = −.276).^211^^,^^222–224^ Postural balance is particularly vulnerable to the effects of sarcopenia. Loss of muscle strength, excessive co-activation of antagonistic groups, joint stiffness, delayed compensatory responses, and early fatigue of stabilizing muscles all contribute to increased sway and reduced stability.^225–227^ Electromyography studies highlight this maladaptation, showing tibialis anterior hyperactivity and premature gluteus maximus fatigue, reflecting reliance on inefficient compensatory strategies.^225^ Large population data confirm the clinical impact: in 3559 older adults (1009 with sarcopenia), the syndrome increased the risk of postural dysfunction by 1.74 times, independent of age and sex.^228^ At the contractile level, single-fiber analyses demonstrate lower maximal isometric force (Po), reduced shortening velocity (Vo), diminished specific tension, and higher passive stiffness in sarcopenic muscle.^229–231^ These deficits are exacerbated by elevated catabolic cytokines and myokines, including myostatin, P3NP, TNF-α, IL-6, and CRP, which further impair force generation and accelerate functional decline.^232^

Bone loss undermines balance, proprioception, and muscle performance. Lower femoral neck/lumbar BMD correlates with worse balance (OR = 0.91; 95% CI 0.874-0.96).^233^ Vertebral deformities and malalignment degrade mechanoreceptor input, trunk/lower-limb position sense, and stabilizing reflexes, increasing fall risk.^234–236^ Individuals with osteoporosis exhibit greater postural sway and joint position errors, evidence of impaired sensorimotor integration.^237^^,^^238^ Although MR studies show mixed direct effects of BMD on walking speed,^239^ osteoporosis tracks with frailty phenotypes that include slower gait.^189^^,^^240^ Given the shared deficits between IC and frailty, it can be hypothesized that osteoporosis adversely impacts the locomotion domain and its subdomains. Mechanistically, high bone turnover elevates transforming growth factor beta (TGF-β), driving oxidative stress in muscle, destabilizing ryanodine receptors, impairing Ca^2+^ handling, and reducing contraction/endurance.^241^^,^^242^ Lower BMD is also causally associated with reduced handgrip strength (*β*  = .01, *p* < .01).^239^ Age-related estrogen/IGF-1 declines and immobilization after fractures compound mobility losses.

When muscle and bone deficits co-occur, locomotor decline is amplified across subdomains. Regarding balance, older adults with sarcopenia plus osteoporosis had significantly worse general, anteroposterior, and mediolateral stability indices than those with sarcopenia or osteopenia alone (*p* < .05).^243^ In 306 fall- and fracture-prone older adults, OS was associated with larger posturography sway areas (eyes open/closed) and higher oscillation velocity on firm surfaces, as well as greater postural instability driven by combined muscle loss, low BMD, proprioceptive deficits, CNS/PNS alterations, frailty, and gait changes.^244^ In 8888 participants from the Canadian Longitudinal Study on Aging, OS (1982 participants) was associated with poorer standing balance (−3.088 seconds) compared with normal BMD/no sarcopenia after full statistical adjustments.^194^ Gait performance is likewise depressed in OS.^14^^,^^109^ With aging, selective type II fiber atrophy reduces hip extensor and plantar-flexor power essential for propulsion^245^; mitochondrial dysfunction and oxidative stress lower ATP production, raising the energetic cost of walking and contributing to concurrent bone loss.^136^^,^^215^

Sarcopenic older people have shorter step/stride, reduced single-support time, and greater AP/lateral variability^220^; osteoporosis adds reduced hip extension in stance, lower hip/ankle power, and altered trunk kinematics,^246^ resulting in lower mechanical efficiency and slower gait. Intramuscular fat infiltration is more pronounced in OS, degrades muscle quality, correlating with slower gait and higher disability risk.^247^^,^^248^ Concurrent proprioceptive impairments further destabilize gait. Strength deficits are robust in OS, exceeding those in isolated sarcopenia.^32^^,^^194^^,^^195^^,^  ^249–251^ Mechanistically, OS couples type II fiber loss and reduced contractile capacity with bone fragility that limits effective force transmission.^14^ Dysregulated mammalian target of rapamycin (mTOR) signaling (muscle protein synthesis) and RANK/RANKL/OPG (bone remodeling) coexist with low-grade inflammation (IL-6, TNF-α), promoting muscle catabolism and bone resorption, thereby reinforcing strength decline.^14^^,^^252^^,^^253^

**Figure 5:**
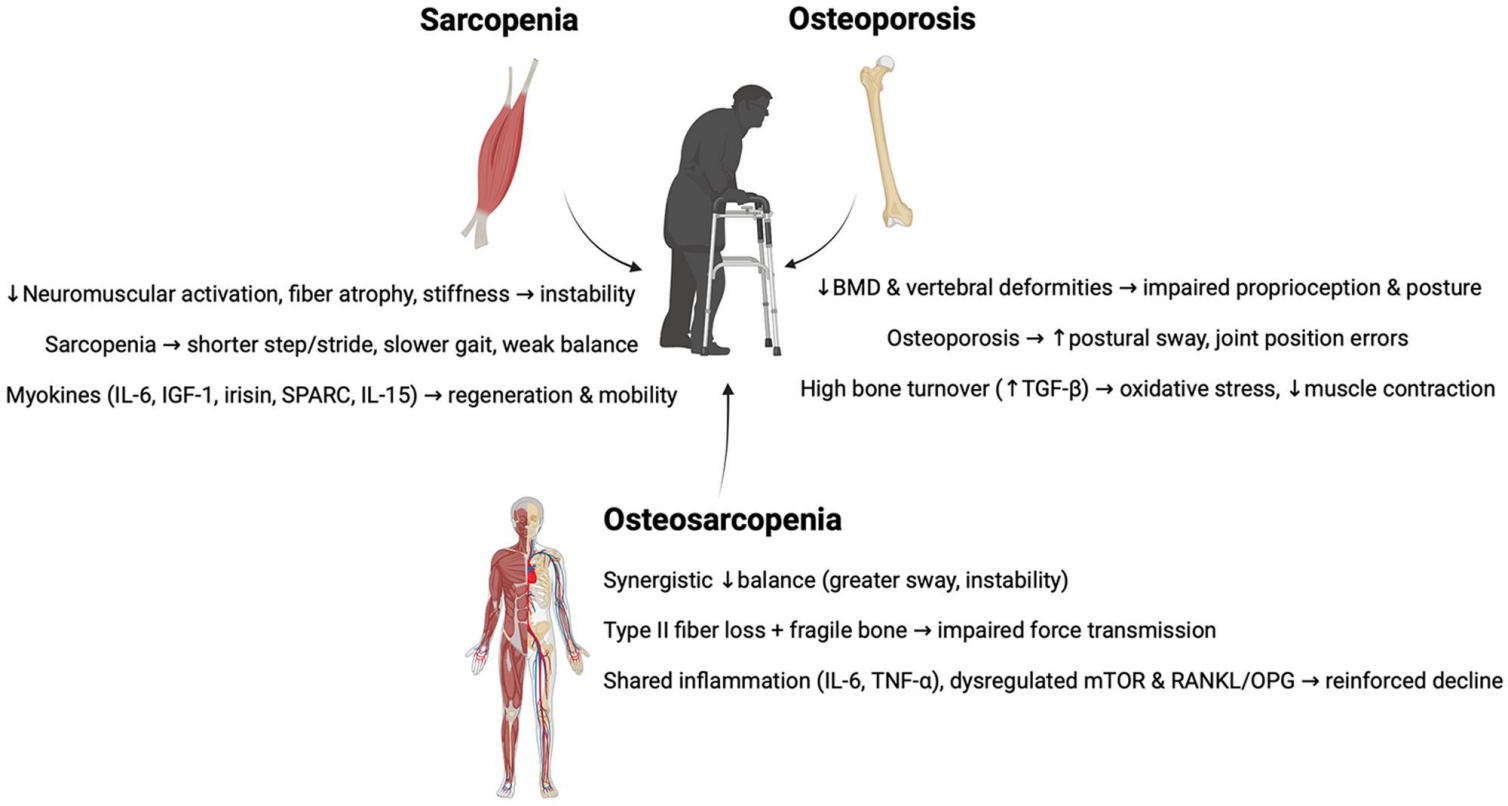
Declines in locomotion domain due to sarcopenia, osteoporosis, and osteosarcopenia. IL-6: interleukin 6; IL-15: interleukin 15; IGF-1: insulin-like growth factor 1; SPARC: secreted protein acidic and rich in cysteine; TGF-β: transforming growth factor beta; mTOR: mammalian target of rapamycin; TNF-α: tumor necrosis factor alpha; RANKL/OPG: receptor activator of nuclear factor kappa-B ligand-osteoprotegerin; BMD: bone mineral density.

In summary, locomotion declines with age due to coordinated impairments across muscle, nerve, and bone. Sarcopenia impairs gait mechanics, balance, and contractile function; osteoporosis disrupts sensorimotor integration, balance, and strength through structural and molecular pathways. Their coexistence as an OS produces greater deficits in balance, gait speed, and strength than either condition alone (Figure 5), underscoring the locomotion domain as a primary IC target for integrated musculoskeletal interventions.

### OS and overall impact on IC

OS exerts a multidimensional impact on IC, with evidence indicating synergistic deterioration across cognitive, psychological, sensory, vitality, and locomotion domains. Among these, locomotion is the most consistently documented domain, whereas cognition, psychological health, sensory function, and vitality remain comparatively understudied, highlighting substantial gaps in the current state of the art.

In the cognitive domain, OS has been strongly associated with cognitive frailty and specific impairments in executive and visuospatial functions, with effect sizes exceeding those observed for sarcopenia or osteoporosis alone. These findings support a synergistic musculoskeletal-neurocognitive axis, likely mediated by shared endocrine, inflammatory, vascular, and neurotrophic pathways. However, longitudinal and mechanistic studies linking OS to dementia trajectories and domain-specific cognitive decline are still limited.

In the psychological domain, emerging longitudinal and cross-sectional evidence indicates that OS increases the risk of depressive symptoms and incident depression, reflecting the combined burden of mobility loss, pain, reduced autonomy, and systemic inflammation. Nevertheless, the causal pathways and bidirectional interactions between emotional health and musculoskeletal degeneration remain insufficiently explored, particularly regarding anxiety, social engagement, and psychological resilience.

The sensory domain represents one of the most critical evidence gaps. Although mechanistic plausibility suggests that OS may exacerbate hearing and visual decline through vascular, metabolic, oxidative, and structural pathways, direct epidemiological evidence remains scarce. Sensory outcomes have rarely been incorporated into OS research, underscoring the need for integrative sensory-musculoskeletal aging frameworks.

Regarding vitality, direct evidence linking OS to metabolic and cardiorespiratory reserve is limited. Nonetheless, converging mechanisms such as chronic inflammation, hormonal dysregulation, nutritional deficits, and vascular impairment suggest that OS likely accelerates declines in energy homeostasis and physiological resilience. Incorporating combined muscle and bone markers into geriatric assessments may improve the identification of individuals at risk for vitality impairment.

The locomotion domain is the most robustly supported IC domain affected by OS. Consistent evidence demonstrates that combined muscle and bone deficits amplify impairments in balance, gait, and strength through neuromuscular, biomechanical, and molecular pathways, resulting in greater functional disability than either condition alone. This reinforces locomotion as a primary target for integrated musculoskeletal interventions within IC frameworks.

Overall, while OS is increasingly recognized as a systemic condition affecting multiple IC domains, substantial knowledge gaps persist in cognition, psychological health, sensory function, and vitality. Future longitudinal and mechanistic studies integrating musculoskeletal, neurocognitive, psychosocial, sensory, and metabolic outcomes are required to clarify causal pathways and inform multidomain interventions aimed at preserving IC in older people.

### Strengths and limitations

This review provides a comprehensive synthesis of current evidence on how musculoskeletal health influences the domains of IC. By examining both the beneficial effects of preserved muscle and bone function and the detrimental consequences of sarcopenia, osteoporosis, and OS, it offers an integrative perspective that, to our knowledge, has not been previously addressed. A key strength is the novelty of this approach, as this is the first review to systematically map the contributions of muscle- and bone-derived mechanisms across all IC domains. Another strength lies in the methodological rigor of the studies included, many of which used validated diagnostic criteria, standardized outcomes, and large population-based cohorts, thereby increasing the reliability and robustness of the evidence summarized. By consolidating these findings, the review establishes a solid platform for future investigations and supports the generation of targeted hypotheses on the complex interplay between musculoskeletal decline and IC in older adults.

At the same time, several limitations must be acknowledged. First, the narrative review design does not provide the same level of rigor, transparency, or reproducibility as systematic reviews, and may introduce subjectivity in study selection and interpretation. Second, the limited number of studies retrieved affected the understanding of the impacts of OS on subdomains of IC, which limits the robustness of the generalization of the evidence compiled in this review. Third, the heterogeneity of study designs, populations, and outcome measures complicates direct comparisons and synthesis. Finally, part of the literature stems from experimental or preclinical models, which offer valuable mechanistic insights but limit direct extrapolation to clinical populations.

These constraints should be considered when interpreting the implications of musculoskeletal health for IC and highlight the need for future longitudinal and interventional studies that can clarify causal pathways and refine strategies to preserve functional capacity in aging.

## Conclusions and future perspectives

The concept of IC provides a comprehensive framework for understanding healthy aging, shifting focus from disease-centered approaches to the preservation of functional abilities. Within this framework, musculoskeletal health emerges as a central determinant of IC. Muscles and bones influence multiple domains not only through biomechanical support but also via their role as secretory organs, releasing myokines and osteokines with endocrine, paracrine, and autocrine actions. Molecules such as BDNF, irisin, uOCN, and anti-inflammatory cytokines directly affect cognition, vitality, psychological resilience, sensory function, and locomotion.

Sarcopenia and osteoporosis, both prevalent yet frequently underdiagnosed in clinical practice, compromise IC beyond mobility, extending their impact to cognition, mood, sensory processing, and overall vitality. Their coexistence as OS amplifies these effects, producing greater deficits in gait, balance, muscle strength, and emotional well-being than either condition alone. Importantly, although OS integrates the coexistence of sarcopenia and osteoporosis, and although sharing very similar risk factors, the biological mechanisms underlying each condition are not fully identical. Sarcopenia is more directly driven by neuromuscular impairment, anabolic resistance, and altered muscle protein turnover and muscle quality, whereas osteoporosis is primarily characterized by impaired bone remodeling, microarchitectural deterioration, and an imbalance between bone formation and resorption. Therefore, OS likely represents a state in which shared pathways converge with additive, condition-specific mechanisms that together exacerbate declines in IC. Accordingly, OS reflects both shared biological mechanisms (e.g., chronic inflammation, hormonal dysregulation, oxidative stress, and metabolic dysfunction) that simultaneously impair bone and muscle, accelerating IC decline.^1^^,^^254^^,^^255^

These findings highlight the importance of early and multidimensional interventions. Structured physical activity, resistance and balance training, adequate protein and micronutrient intake, and targeted pharmacological strategies hold strong potential for mitigating decline across IC domains. Equally, clinical practice should incorporate simple, validated tools, such as HGS, gait speed, and BMD, as early indicators of IC deterioration. Future work should prioritize biomarker discovery and validation, particularly involving myokines, osteokines, and inflammatory mediators, to refine risk stratification and guide interventions.

Despite growing evidence, key gaps remain. Few longitudinal or interventional studies have directly examined how sarcopenia, osteoporosis, and OS affect IC trajectories across diverse populations. Future research should therefore focus on clarifying causal pathways, developing standardized IC assessments in clinical and community settings, and testing integrated interventions that simultaneously target muscle and bone. Addressing these gaps will be critical to translate the concept of IC into practice, enabling more precise, preventive, and person-centered strategies to extend functional independence and quality of life in aging populations worldwide.

## Data Availability

No new data were created or analyzed in this study. Data sharing is not applicable to this article.
